# TAFRO Syndrome in a Kidney Transplant Recipient That Was Diagnosed on Autopsy: A Case Report

**DOI:** 10.3389/fmed.2021.747678

**Published:** 2021-10-04

**Authors:** Marie Nagai, Takahiro Uchida, Muneharu Yamada, Shuuhei Komatsu, Kohei Ota, Mitsuya Mukae, Hitoshi Iwamoto, Hiroshi Hirano, Miho Karube, Shinya Kaname, Takashi Oda

**Affiliations:** ^1^Department of Nephrology and Blood Purification, Kidney Disease Center, Tokyo Medical University Hachioji Medical Center, Hachioji, Japan; ^2^Department of Kidney Transplantation Surgery, Kidney Disease Center, Tokyo Medical University Hachioji Medical Center, Hachioji, Japan; ^3^Departmet of Diagnostic Pathology, Tokyo Medical University Hachioji Medical Center, Hachioji, Japan; ^4^Department of Nephrology and Rheumatology, Kyorin University School of Medicine, Tokyo, Japan

**Keywords:** autopsy, kidney transplant, membranoproliferative glomerulonephritis, TAFRO syndrome, thrombotic microangiopathy

## Abstract

A 57-year-old man who received a kidney transplant 4 years previously owing to unknown underlying disease presented with thrombocytopenia and fever. Hepatosplenomegaly and lymphadenopathy were observed, and development of prominent anasarca and worsening of renal function yielded the diagnosis of TAFRO syndrome. He was treated with high-dose steroids and plasmapheresis, and a thrombopoietin receptor agonist was administered for refractory thrombocytopenia. However, his general condition worsened, and he died on day 92. Histopathological analysis of a kidney autopsy specimen showed thrombotic microangiopathy characterized by glomerular endothelial swelling, mesangiolysis, and double contours of the glomerular capillary walls. His bone marrow showed megakaryocytic hyperplasia with mild reticulin fibrosis. Interestingly, these clinical and pathological features were remarkably similar to those the patient demonstrated before the kidney transplant, suggesting the recurrence of TAFRO syndrome. TAFRO syndrome is a rare systemic disorder whose concept has recently been established, but information on its long-term outcome is scarce. To our knowledge, this is the first case of TAFRO syndrome developing in a kidney transplant recipient, which suggests that disease recurrence occurs many years after the kidney transplant.

## Introduction

TAFRO syndrome is a systemic inflammatory disorder characterized by thrombocytopenia, anasarca, fever, renal dysfunction, myelofibrosis, and organomegaly (1). This disorder often has an acute/subacute onset, and the involvement of hypercytokinemia, such as increased levels of interleukin (IL)-6 and vascular endothelial growth factor, which may be triggered by undetected infections (2), is hypothesized in its pathogenesis (3). This is the syndrome of heterogeneous entity; although several diagnostic criteria have been proposed (2, 4), the precise disease mechanism common to this syndrome still remains to be fully understood.

It has been known that this rare clinical entity can occur in association with idiopathic multicentric Castleman disease (iMCD) which presents systemic inflammation and multifocal lymphadenopathy accompanied by characteristic histopathological features. iMCD patients with TAFRO syndrome (iMCD-TAFRO) is now considered to be an aggressive clinical subtype of iMCD (5), and new definition that focuses on histopathological findings of lymph node and categorizes the affected cases into iMCD-TAFRO and TAFRO syndrome without iMCD has been proposed very recently (6).

Many case reports of TAFRO syndrome have been published since it was originally reported in 2010. Renal dysfunction is reportedly observed in more than one-half of patients with this disorder (7) and can be a life-threatening complication, because the degree of renal dysfunction is often very severe and not a few patients require renal replacement therapy (RRT) (8, 9). However, in most cases, discontinuation of RRT is possible after effective treatment, and therefore few patients require maintenance dialysis or a kidney transplant.

We herein report an autopsy case of a kidney transplant recipient with unknown underlying disease, who developed refractory TAFRO syndrome 4 years after the transplant. In the present case, the clinical and pathological features observed before and after the kidney transplant were very similar, suggesting the recurrence of TAFRO syndrome.

## Case Presentation

**Figure 1:**
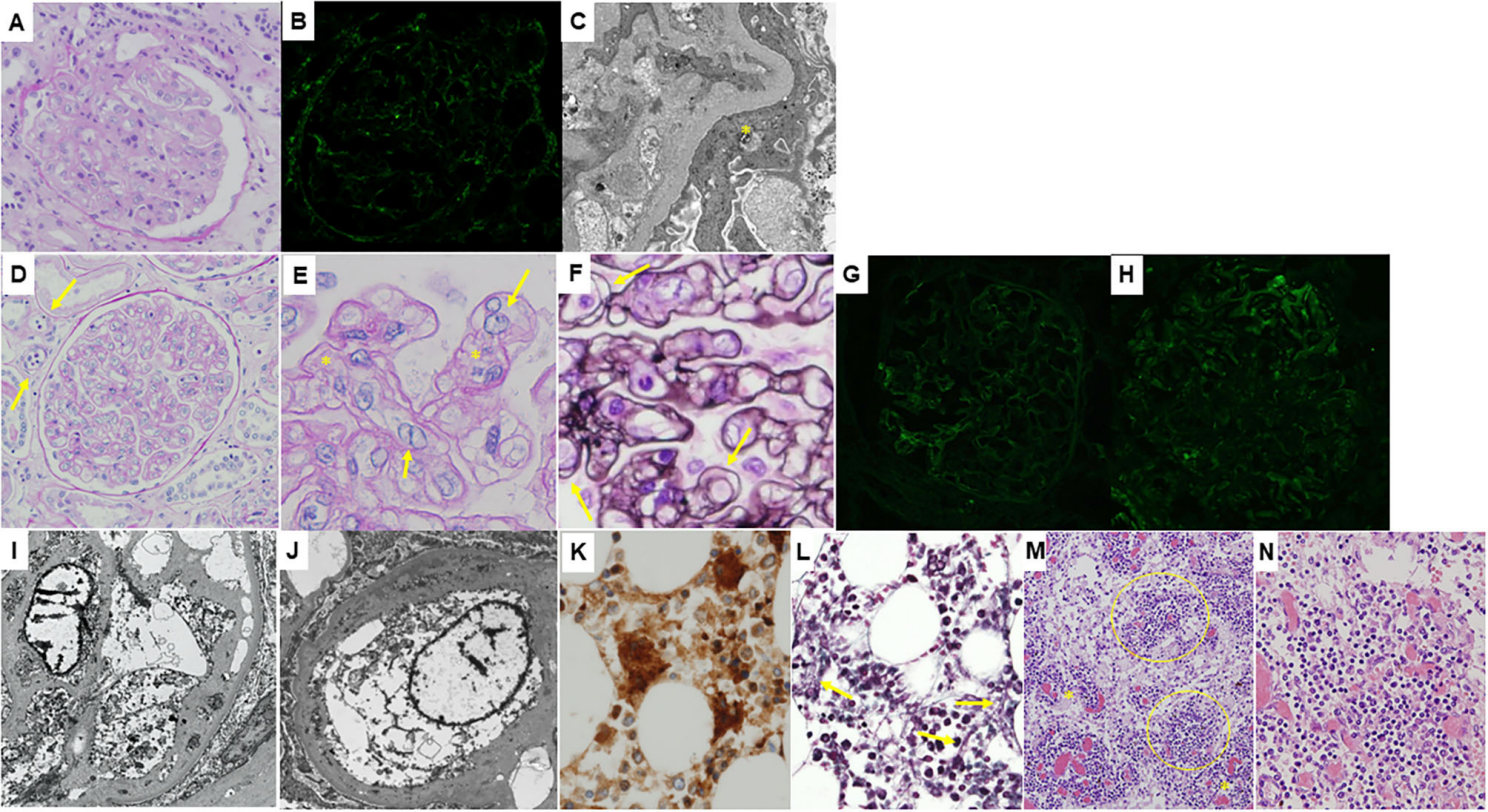
Histological features of the patient. **(A–C)**: Microphotographs of the native kidney. **(A)** Light microscopy image of a periodic acid-Schiff-stained section showing mesangial proliferation and thickening of the glomerular capillary walls, i.e., membranoproliferative glomerulonephritis pattern. Swelling of glomerular endothelial cells is also seen. **(B)** Immunofluorescence staining for immunoglobulin G showing only faint deposition in the glomeruli. **(C)** An electron microscopy section showing no obvious electron-dense deposits. Podocyte foot process effacement is shown (yellow asterisk). **(D–N)**: Microscopic findings of the autopsy samples. **(D)** Light microscopy image of a periodic acid-Schiff-stained transplanted kidney section showing prominent swelling of glomerular endothelial cells, mesangiolysis, and duplication of the glomerular capillary walls suggesting thrombotic microangiopathy. Peritubular capillaritis is also shown (yellow arrows). A higher magnification image of a periodic acid-Schiff-stained transplanted kidney section demonstrating endothelial swelling (yellow arrows) as well as mesangiolytic lesions (yellow asterisks, **E**) and a periodic acid-methenamine-silver-stained transplanted kidney section clearly showing duplication of the glomerular capillary walls (yellow arrows, **F**). Immunofluorescence staining showing weak deposition of immunoglobulin G **(G)** and complement C3 **(H)** in the glomeruli. Electron microscopy sections showing swelling of glomerular endothelial cells and double contours of the glomerular basement membrane **(I)** and widening of the subendothelial space **(J)**. Immunoperoxidase staining for CD42b of the bone marrow tissue showing an increase in megakaryocytes **(K)**. Silver staining of the bone marrow tissue demonstrating mild reticulin myelofibrosis (yellow arrows in **L**). **(M)** Atrophic germinal centers (yellow circles) with proliferation of the dilated capillaries in the interfollicular areas (yellow asterisks) were seen in the mildly enlarged lymph nodes (hematoxylin and eosin stain). A higher magnification image of the lymph node showing germinal centers penetrated by hyalinized vessels (hematoxylin and eosin stain, **N**).

A 57-year-old man who underwent a kidney transplant 4 years previously presented with persistent fever and abdominal back pain, and was admitted to our hospital. Seventeen years previously, he was suspected of having systemic lupus erythematosus (SLE) owing to thrombocytopenia, ascites, and proteinuria, as well as serological positivity for the antinuclear antibody (1:160) and anti-double-stranded (anti-ds) DNA antibody (27.6 IU/mL). However, these autoantibodies became negative soon and were consistently negative thereafter, and hypocomplementemia was never observed. In addition, renal biopsy showed membranoproliferative glomerulonephritis (MPGN) pattern of injury with few immune deposits (Figures 1A–C). Thus, the etiology of his original kidney disease was rather unclear. His bone marrow demonstrated mild myelofibrosis with increased megakaryocytes (data not shown). His renal function gradually deteriorated, and maintenance hemodialysis was initiated 5 years previously. One year after the start of hemodialysis, he received a living-donor kidney transplant. He remained well after the transplant, and was treated with tacrolimus, mycophenolate mofetil, and prednisone at 1 mg daily.

On physical examination, his body temperature was 37.3°C and blood pressure was 132/73 mmHg. Abdominal distension, leg edema, and swelling of the submandibular lymph nodes were observed. His laboratory test results are summarized in Table 1. Urinalysis showed dysmorphic hematuria and nephrotic-range proteinuria. Decoy cells that suggest BK virus infection were never found in the repeated examination of his urine. Blood analysis showed severe thrombocytopenia (platelet count: 2.3 × 10^4^/μL), but hemolytic anemia was absent (the level of lactate dehydrogenase was not elevated and that of haptoglobin was not decreased). Decrease in a disintegrin-like and metalloproteinase with thrombospondin type 1 motifs 13 (ADAMTS13) activity was not seen, and abnormalities of the blood coagulation system to suggest disseminated intravascular coagulation were scarce. Levels of transaminase were normal, but his alkaline phosphatase level was substantially increased. Immunoglobulin (Ig) levels were slightly decreased, but immunoelectrophoresis did not detect any monoclonal proteins. Levels of C-reactive protein and IL-6 were increased, but there were no signs of viral infection, including Epstein-Barr virus or cytomegalovirus, and levels of both complements C3 and C4 were within the normal range. The antinuclear antibody and disease-specific autoantibodies, such as the anti-dsDNA antibody and anti-neutrophil cytoplasmic antibody were negative, and donor-specific antibodies were not detected. Computed tomography displayed pericardial effusion, pleural effusion, massive ascites, retroperitoneal lymphadenopathy, and hepatosplenomegaly, but there was no evidence of malignant solid tumors or signs of infection.

**Table 1:** Laboratory data of the patient.

**Urinalysis**		**Serology**	
Occult blood	2+	IgG	632 mg/dL
Protein	3+	IgA	243 mg/dL
RBC	30–49/HPF	IgM	23 mg/dL
WBC	1–4/HPF	Complement C3	90.1 mg/dL
Proteinuria in 24-h urine	4.2 g/day	Complement C4	17.2 mg/dL
		ANA titer	<40
**Complete blood count**		dsDNA-IgG	<10 IU/mL
WBC	3,980/μL	anti-SS-A/Ro antibody	<1.0 U/mL
Hemoglobin	11.6 g/dL	anti-SS-B/La antibody	<1.0 U/mL
Platelet	23,000/μL	MPO-ANCA	0.0 U/mL
		PR3-ANCA	0.1 U/mL
**Biochemistry**		Haptoglobin	185 mg/dL
Creatinine	0.98 mg/dL	ADAMTS13 activity	25%
Blood urea nitrogen	19.2 mg/dL	Soluble IL-2 receptor	1,036 U/mL
Total protein	6.1 g/dL	IL-6	19.0 pg/mL
Albumin	3.2 g/dL	HBs antigen	Negative
Aspartate aminotransferase	28 U/L	HCV antibody	Negative
Alanine aminotransferase	28 U/L	HIV antibody	Negative
Lactate dehydrogenase	143 U/L		
Total bilirubin	0.9 mg/dL	**Coagulation**	
Alkaline phosphatase	1015 U/L	PT-INR	1.02
CRP	6.1 mg/dL	Fibrinogen	494 mg/dL
		FDP	11.1 μg/mL

*ADAMTS13, a disintegrin-like and metalloproteinase with thrombospondin type 1 motifs 13; ANA, antinuclear antibody; ANCA, anti-neutrophil cytoplasmic antibody; CRP, C-reactive protein; dsDNA, double-stranded DNA; FDP, fibrinogen degradation products; HBs, hepatitis B surface; HCV, hepatitis C virus; HIV, human immunodeficiency virus; HPF, high-power field; Ig, immunoglobulin; IL, interleukin; MPO, myeloperoxidase; PR3, proteinase 3; PT-INR, prothrombin time-international normalized ratio; RBC, red blood cell; WBC, white blood cell*.

After admission, his anasarca worsened, and his renal function rapidly deteriorated. Autoimmune disorders such as Sjogren's syndrome or anti-neutrophil cytoplasmic antibody-associated vasculitis were unlikely because antinuclear antibody and disease-specific autoantibodies were negative. There was no hemolytic anemia or hypocomplementemia, and atypical hemolytic uremic syndrome was also unlikely. Decrease in ADAMTS13 activity was absent, and the possibility of thrombotic thrombocytopenic purpura was excluded. Computed tomography scan did not show evidence of malignancies or infectious disorders. Thus, typical clinical features (thrombocytopenia, anasarca, systemic inflammation, organomegaly, and progressive renal dysfunction) and the exclusion of differential diagnoses led to the diagnosis of TAFRO syndrome. In addition, the clinical course was very similar to that prior to the kidney transplant. Two courses of intravenous methylprednisolone pulse therapy were performed, and the daily dose of steroids was increased, which temporarily resulted in the improvement of renal function and a decrease in C-reactive protein level. However, the patient remained in a condition of transfusion-dependent severe thrombocytopenia, and therefore the administration of romiplostim (a thrombopoietin receptor agonist) was initiated. In addition, five sessions of plasmapheresis using fresh frozen plasma were performed. Although the patient's platelet count increased to some extent by these treatments, it robustly decreased soon after the termination of plasmapheresis, followed by the deterioration of renal function and an increase in C-reactive protein level again. Although a third course of intravenous methylprednisolone pulse therapy was performed, the patient died on day 92 of hospitalization after complaining of chest discomfort.

Consent for autopsy was obtained from the patient's family. The transplanted kidney weighed 420 g, and microscopically showed thrombotic microangiopathy (TMA): there were prominent swelling of glomerular endothelial cells, mesangiolysis, and duplication of the glomerular capillary walls (Figures 1D–F). Thrombi were not found in the glomeruli or peritubular capillaries. Immunofluorescence staining showed focal deposition of Igs and complements (Figures 1G,H). Electron microscopy sections demonstrated glomerular endothelial swelling and widening of the subendothelial space, as well as double contours of the glomerular basement membrane (Figures 1I,J). The bone marrow showed mild hyperplasia with mild reticulin fibrosis, and there was an increase in megakaryocytes (Figures 1K,L). Several lymph nodes were mildly enlarged, and atrophic germinal centers with proliferation of the dilated capillaries in the interfollicular areas were observed (Figure 1M). Germinal centers were penetrated by hyalinized vessels, but neither atypical lymphocytes nor plasma cells were found (Figure 1N). These features of lymph nodes were compatible with hypervascular type of iMCD (10).

Other notable findings were as follows. There was massive ascites and pleural effusion. The spleen weighed 410 g, and showed features of congestion. There were no signs suggesting viral infection or acute cardiovascular events, such as myocardial infarction or aortic dissection. From these autopsy findings, a final diagnosis of TAFRO syndrome was made.

## Discussion

**Figure 2:**
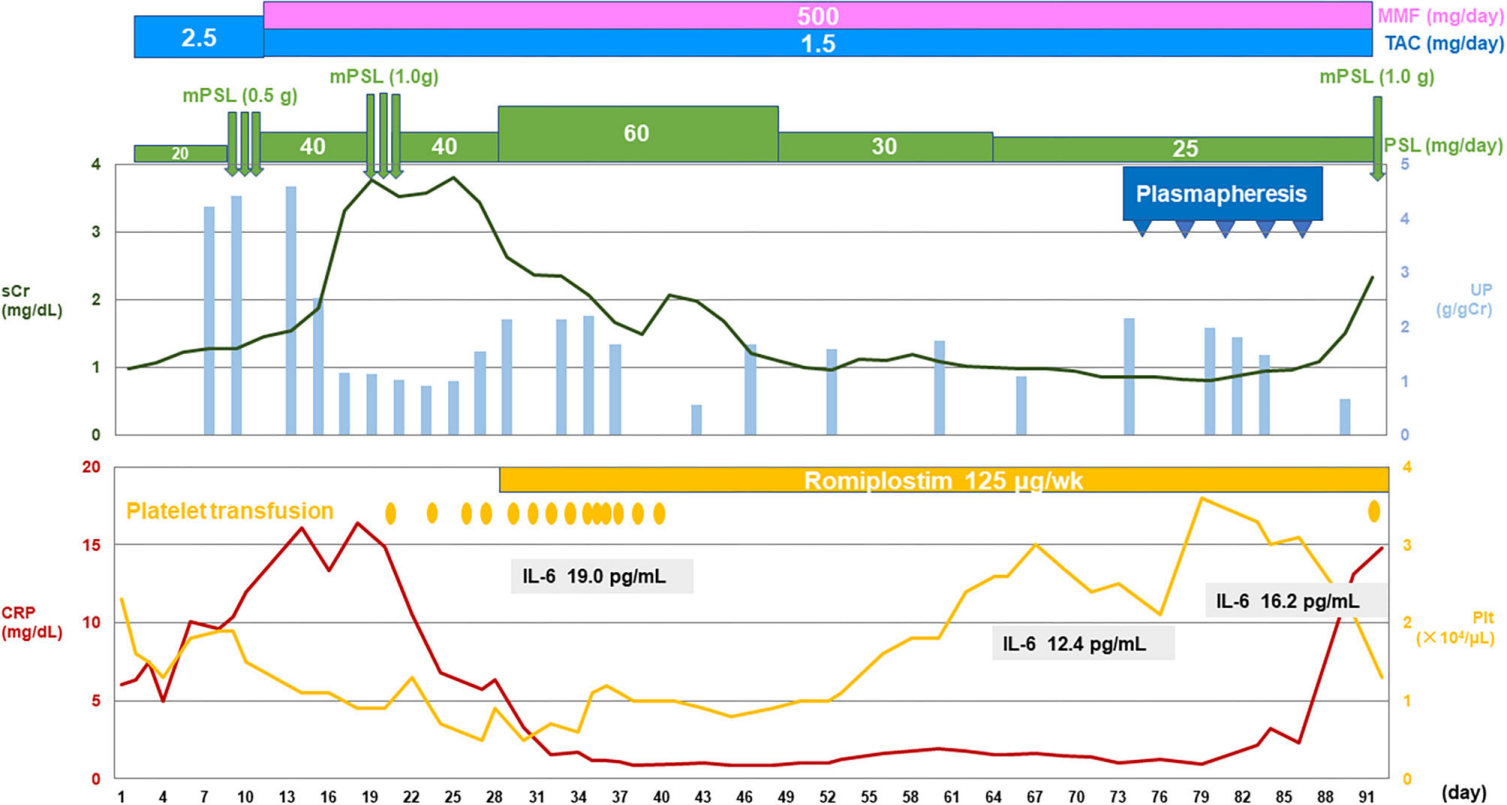
The patient's clinical course.CRP, C-reactive protein; IL, interleukin; MMF, mycophenolate mofetil; mPSL, methylprednisolone; Plt, platelet; sCr, serum creatinine; TAC, tacrolimus; UP, urinary protein.

To our knowledge, this is the first reported case of TAFRO syndrome developing in a kidney transplant recipient. Based on the recently published definition (6), the present case might be categorized into iMCD-TAFRO; however, the definition had not been clearly proposed when we treated the patient. We therefore diagnosed him simply as TAFRO syndrome according to the diagnostic criteria of the day (4). His clinical course is shown in Figure 2. Because the patient fulfilled the classification criteria of SLE (11, 12) at the time of disease onset, and because the disease concept of TAFRO syndrome had not yet been established, the exclusion of SLE was difficult at that time. However, we considered that SLE was unlikely because of the following reasons: (1) the titers of serum antinuclear antibody and anti-dsDNA antibody were 1:160 and 27.6 IU/mL, respectively, which were not so high, and both titers became negative in the next month and were consistently negative thereafter, (2) hypocomplementemia was never observed, and (3) renal biopsy did not show full-house immune deposits, but only few immune deposits. On the other hand, as summarized in Table 2, the clinical and pathological features observed both before and after the kidney transplant were similar, which suggested recurrent TAFRO syndrome. We performed an English literature search but could not find any previously reported cases of recurrent TAFRO syndrome. Thus, this case is highly suggestive, in which the patient's long-term clinical course and histological features, including changes in both the native and transplanted kidney were observed.

**Table 2:** Summary of clinical and pathological features of the patient.

	**13 years prior to kidney transplant**	**4 years after kidney transplant**
Diagnostic criteria^*1^		
Major categories		
Thrombocytopenia	Yes	Yes
Anasarca	Yes	Yes
Systemic inflammation	Yes	Yes
Minor categories		
Lymph node findings	Not sampled	Atypical
Bone marrow findings	Typical	Typical
Mild organomegaly	Yes	Yes^*2^
Progressive renal dysfunction	Yes	Yes
Other notable clinical features	Transient serological positivity of ANA and anti-dsDNA Ab	Increased serum ALP level with normal serum LDH level
Major pathological findings		
Kidney	MPGN pattern of injury with few immune deposits	TMA
Bone marrow	Increase in megakaryocytes and mild reticulin fibrosis	Increase in megakaryocytes and mild reticulin fibrosis
Treatment		
Immunosuppressive therapy	Steroid and cyclosporine	Steroid, tacrolimus, and mycophenolate mofetil
Blood purification therapy	Immunoadsorption	Plasmapheresis
Thrombopoietin receptor agonist	No	Yes
Prognosis	End-stage renal disease	Death

*Ab, antibody; ALP, alkaline phosphatase; ANA, antinuclear antibody; dsDNA, double-stranded DNA; LDH, lactate dehydrogenase; MPGN, membranoproliferative glomerulonephritis; TMA, thrombotic microangiopathy*.

*1 * Based on the 2019 updated diagnostic criteria for TAFRO syndrome by Masaki et al. (4)*.

*2 * Although mild hepatomegaly and lymphadenopathy were observed, massive splenomegaly with features of congestion was also observed*.

Severe renal dysfunction is frequently observed in patients with TAFRO syndrome, and not a few patients require RRT. However, the discontinuation of RRT is reportedly possible in the majority of patients; Mizuno et al. (9) reported that hemodialysis was initiated in 4 out of 7 patients, but was discontinued in all of them. Leurs et al. (8) reported that nine out of 19 patients (including two of the patients reported by Mizuno et al.) required hemodialysis, but only one patient needed maintenance hemodialysis (14). Some types of bacterial infection are supposed to be involved in the pathogenesis of TAFRO syndrome (2, 13), and the recurrence of disease in this patient may also be triggered by infections. However, detailed examination of our patient did not show any evidence of infection. Another possible explanation for the recurrence is that the maintenance immunosuppressive therapy was too mild for the suppression of disease activity.

Light microscopic renal histology findings in patients with TAFRO syndrome have been mainly limited to two patterns: TMA-like glomerulopathy and MPGN-like glomerulopathy (3, 8). In this regard, it has been suggested that TMA-like lesions are seen in the acute phase and MPGN-like lesions are seen in the chronic phase as a result of the TMA-like lesions, and that both lesions are continuous renal pathological conditions in TAFRO syndrome, which may involve glomerular endothelial cell injury and macrophage infiltration (8, 15). In accordance with these previous reports, native kidney biopsy of the present patient showed MPGN pattern of injury with few immune deposits, whereas an autopsy specimen of the transplanted kidney, which was obtained in the acute phase of the disease, showed TMA (Table 2).

Most patients with TAFRO syndrome receive steroid therapy as the first-line treatment. Rituximab (an anti-CD20 antibody), tocilizumab (an anti-IL-6 receptor antibody), and cyclosporine are commonly used in steroid-resistant patients, and a recent study in Japan proposed that rituximab was a promising agent (16). In this regard, it would be reasonable to modify the therapeutic strategy according to the primary disease process, because the process is T cell-dominant in some patients but is possibly plasma cell-dominant in others. However, our patient had been treated with tacrolimus and mycophenolate mofetil in addition to steroids for many years after the kidney transplant, and hence the excessive immunosuppressed state that would be caused by the additional administration of rituximab or tocilizumab was of great concern. Plasmapheresis was performed for the rapid removal of humoral factors that are involved in disease pathogenesis (17). Indeed, the platelet count of our patient was increased to a certain extent, but it robustly decreased soon after termination of plasmapheresis.

In conclusion, we reported the first case of TAFRO syndrome in a kidney transplant recipient. This case suggests that its recurrence occurs many years after the kidney transplant. The further accumulation of cases is needed to investigate the appropriate treatment strategies, including RRT, long-term outcomes, and recurrence of this disorder.

## Data Availability Statement

The original contributions presented in the study are included in the article/supplementary material, further inquiries can be directed to the corresponding author/s.

## Ethics Statement

Written informed consent was obtained from the patient's family for the publication of any potentially identifiable images or data included in this article.

## Author Contributions

MN and TU: writing the manuscript draft. TO: critical manuscript revision. MN, TU, SK, KO, MM, HI, and MK: clinical care of the patient. TU, MY, HH, SK, and TO: histological evaluation. All authors contributed to the article and approved the submitted version.

## Conflict of Interest

The authors declare that the research was conducted in the absence of any commercial or financial relationships that could be construed as a potential conflict of interest.

## Publisher's Note

All claims expressed in this article are solely those of the authors and do not necessarily represent those of their affiliated organizations, or those of the publisher, the editors and the reviewers. Any product that may be evaluated in this article, or claim that may be made by its manufacturer, is not guaranteed or endorsed by the publisher.
